# Cappable-Seq Reveals Specific Patterns of Metabolism and Virulence for *Salmonella* Typhimurium Intracellular Survival within *Acanthamoeba castellanii*

**DOI:** 10.3390/ijms22169077

**Published:** 2021-08-23

**Authors:** Alexander S. Balkin, Andrey O. Plotnikov, Natalia E. Gogoleva, Yuri V. Gogolev, Kirill N. Demchenko, Sergey V. Cherkasov

**Affiliations:** 1 Laboratory of Biomedical Technologies, Institute for Cellular and Intracellular Symbiosis, Ural Branch of Russian Academy of Sciences, 460000 Orenburg, Russia; cherkasovsv@yandex.ru; 2 “Persistence of Microorganisms” Science Resource Center, Institute for Cellular and Intracellular Symbiosis, Ural Branch of Russian Academy of Sciences, 460000 Orenburg, Russia; protoz@mail.ru (A.O.P.); negogoleva@gmail.com (N.E.G.); gogolev@kibb.knc.ru (Y.V.G.); 3 Federal Research Center Kazan Scientific Center of the Russian Academy of Sciences, Kazan Institute of Biochemistry and Biophysics, 420111 Kazan, Russia; 4 Laboratory of Cellular and Molecular Mechanisms of Plant Development, Komarov Botanical Institute, Russian Academy of Sciences, 197376 Saint Petersburg, Russia; Demchenko@binran.ru

**Keywords:** *Acanthamoeba*, *Salmonella*, protozoa, Cappable-Seq, glyoxylate cycle, oxidative stress, SPI, persistence

## Abstract

The bacterial pathogen *Salmonella enterica*, which causes enteritis, has a broad host range and extensive environmental longevity. In water and soil, Salmonella interacts with protozoa and multiplies inside their phagosomes. Although this relationship resembles that between *Salmonella* and mammalian phagocytes, the interaction mechanisms and bacterial genes involved are unclear. Here, we characterized global gene expression patterns of *S. enterica* serovar Typhimurium within *Acanthamoeba castellanii* at the early stage of infection by Cappable-Seq. Gene expression features of *S.* Typhimurium within *A. castellanii* were presented with downregulation of glycolysis-related, and upregulation of glyoxylate cycle-related genes. Expression of *Salmonella* Pathogenicity Island-1 (SPI-1), chemotaxis system, and flagellar apparatus genes was upregulated. Furthermore, expression of genes mediating oxidative stress response and iron uptake was upregulated within *A. castellanii* as well as within mammalian phagocytes. Hence, global *S.* Typhimurium gene expression patterns within *A. castellanii* help better understand the molecular mechanisms of *Salmonella* adaptation to an amoeba cell and intracellular persistence in protozoa inhabiting water and soil ecosystems.

## 1. Introduction

Salmonellosis is the second-most common human zoonosis after *Campylobacter* infection, despite a significant decrease in the number of confirmed cases in the EU/EEA since 2008 [1]. In 2017, there were 91,662 confirmed cases of salmonellosis in Europe [1].

In natural reservoirs, *Salmonella* strains may share their habitat with phagocytic protozoa [2,3] that use bacteria as food [4,5] and engulf them by phagocytosis [6,7]. However, *Salmonella* can survive [8,9,10] and replicate within protozoan phagosomes [11,12]. For this reason, certain protozoa are environmental reservoirs for bacterial pathogens. Multiplication of *Salmonella* in protozoan phagosomes increase bacterial virulence [13], enhance their resistance, and enable them to persist in mammalian phagocytes [5,14]. Therefore, the study of the interactions between *Salmonella* and protozoa is crucial for control human and animal salmonellosis.

*Salmonella* survival in mammalian phagocytes and nonphagocytic cells has been investigated with omics technologies [15,16,17,18,19,20]. Within mammalian phagocytes gene expression of the type III secretion system (T3SS), iron uptake and oxidative stress response is strongly upregulated. However, expression of bacterial flagellar and chemotaxis genes is downregulated inside mammalian phagocytes and nonphagocytic cells [15,17,19,20].

The transcriptional profile of *Salmonella* within mammalian phagosomes is well described. Nevertheless, bacterial gene expression in protozoan *Salmonella*-containing vacuoles (SCV) remains unclear. qRT–PCR analyses have disclosed that the expression of *Salmonella* Pathogenicity Islands (SPI) genes was similar in both protozoan SCV and mammalian macrophages [21]. In *Acanthamoeba polyphaga* phagosomes, SPI-2 genes were upregulated [21] whereas SPI-1 genes were not. Only two studies were published on total gene expression in *Salmonella* engulfed by heterotrophic protists such as the ciliate *Tetrahymena sp.* and the amoeba *Acanthamoeba rhysodes* [14,22]. Microarray methods revealed that several genes required for *Salmonella* survival and replication in mammalian phagocytes were also upregulated in protozoa [14,22]. Analysis of the host–pathogen interactions between wild-type or mutant *S.* Typhimurium and the social amoeba *Dictyostelium discoideum* showed that aromatic compounds, lipopolysaccharides (LPSs), O-antigen, SPI-1, SPI-2, SPI-6, and PhoP/PhoQ are required for *Salmonella* to survive within protozoa [12].

Despite the major ecological and epidemiological significance of *Salmonella*, its transcriptome and metabolism in protozoan phagosomes are poorly understood because of the limitations of the methods used and the high costs associated with them. Protozoa are rarely used as model organisms. Consequently, commercially available solutions for cDNA libraries provide inefficient removal of ribosomal and transport protist RNA. Nevertheless, new methods such as dRNA-Seq [23] and Cappable-Seq [24] generate RNA-Seq libraries with mRNA-enriched transcripts. To the best of our knowledge, *Salmonella* gene expression within protozoan phagosomes has not yet been investigated by next-generation sequencing (NGS).

The aim of our study was to reveal specific changes of metabolism and virulence for intracellular survival of *S.* Typhimurium within *Acanthamoeba castellanii* using Cappable-Seq. Additionally, we compared the transcriptional profile of *Salmonella* located within *Acanthamoeba* against those for *Salmonella* within macrophages, epithelial cells, and fibroblasts. Cappable-Seq provided new data for the global evaluation of the *Salmonella* primary transcriptome. We found that expression of genes mediating oxidative stress response and iron uptake was upregulated within *A. castellanii* as well as within mammalian phagocytes. Unlike infection models of macrophages and epithelial cells, *Salmonella* in *Acanthamoeba* SCV at the early stage of infection presented with specific features, such as downregulation of glycolysis-related genes and upregulation of glyoxylate cycle-related, SPI-1, chemotaxis system, and flagellar apparatus genes. Besides, expression of SPI-2 genes was unaltered, like recently shown in a fibroblast infection model [20]. These observations help explain the ability of *Salmonella* to survive inside protozoa and persist in the environment.

## 2. Results

### 2.1. Sequencing

A total of >71 million single-end reads were generated and represented six libraries under two culture conditions, namely *Salmonella* in *Acanthamoeba* SCV (SA) vs. *Salmonella* (S) in the absence of the protists (control). Over 99% reads of the control libraries were mapped to the *S. enterica* serovar Typhimurium 14028S genome (GCA_000022165.1). Most of the library reads for the SA cultures (75–92%) were mapped to the *A. castellanii* genome (GCA_000313135.1). The proportion of reads mapped to the *Salmonella* genome was in the range of 8–15% (Table S1).

There were 571 operons (DEOs) that significantly differentially expressed (FDR-corrected *p* = 0.05; minimum normalized read count = 10; log_2_ fold change > 1) in *Salmonella* within *Acanthamoeba* compared to control culture (Table S3). In the SA libraries, 289 operons were upregulated and 282 were downregulated. From 571 differentially expressed operons, 122 operons had unknown functions. Of these, 87 were downregulated and 35 were upregulated.

### 2.2. Transcriptional Profile of Salmonella within the Acanthamoebae Phagosomes

**Figure 1:**
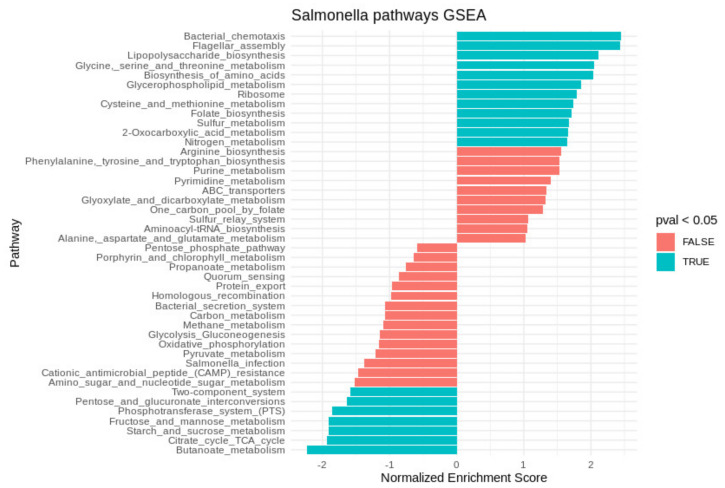
Gene set enrichment analysis performed using the fgsea package. Positive (normalized) enrichment scores indicate upregulation of genes in the pathway in *Salmonella* within *Acanthamoeba*. Negative (normalized) enrichment scores indicate downregulation of genes in the pathway in *Salmonella* within *Acanthamoeba*. Operon pathway annotation was determined against the KEGG database.

In intracellular *Salmonella,* most of the operons with upregulated expression belonged to bacterial chemotaxis and flagellar assembly, LPS biosynthesis, glycerophospholipid metabolism, amino acid biosynthesis, and glyoxylate and dicarboxylate metabolism, according to KEGG (Kyoto Encyclopedia of Genes and Genomes) (Figure 1). Expression of the operons belonging to the tricarboxylic acid cycle (TCA), fructose and mannose metabolism, pyruvate and butanoate metabolism, oxidative phosphorylation, and phosphotransferase system was downregulated (Figure 1).

Numerous operons belonging to “carbohydrate transport and metabolism” from COG (Cluster of Orthologous Groups) had increased expression within *Acanthamoeba*. Expression of operons involved in intracellular trafficking processes, secretion, vesicular transport, replication, recombination, and repair were significantly upregulated. Expression of genes controlling translation, ribosomal structure and biogenesis, posttranslational modification, protein turnover, chaperone synthesis, and energy production and conversion were downregulated.

### 2.3. Expression of Genes Associated with Iron Uptake and Stress Response

Expression of the SPI-1-encoded operon *sitABCD* responsible for iron uptake was upregulated within *Acanthamoeba* (Table 1) as were the operons not located in the SPI regions. Hence, the operons *sufABCDSE*, *fepAE*, *entS*, *entD*, *fepB*, and *ybdA* were activated > 2× within *Acanthamoeba* compared to those in the control libraries (Table 1), as well as the aluminum-inducible gene *STM14_2833* (*ais*), which is activated under iron depletion [25]. However, expression of several operons involved in iron uptake were downregulated in the *Salmonella* within *Acanthamoeba* SCV, including operons of iron transporter *feoAB*, the fumarate reductase flavoprotein *frdABCD*, and the iron storage and detoxification protein bfr.

Here, several genes involved in zinc homeostasis were differentially expressed. The genes of zinc transporter ATPase (*zntA*), the zinc-responsive two-component regulator (*zraSR*), and the putative zinc-dependent peptidase (*yhjJ*) were upregulated, whereas *yggG* (zinc-dependent protease) and *zraP* (*zraSR* two-component system repressor) were downregulated.

Expression of genes encoding oxidative stress response glutaredoxin (*grxA*), the hydroperoxidase (*katG*), the putative oxidoreductases (*STM14_4644*, *STM14_4645*, and *yieEF*), the superoxide dismutase *sodA*, the putative thiol-alkyl hydroperoxide reductase (*STM14_0476*), the alkyl hydroperoxide reductase (*ahpCF*), and the putative iron-dependent peroxidases (*STM14_3004* and *STM14_3005*) was upregulated within *Acanthamoeba* (Table 1). In contrast, expression of the superoxide dismutases genes *sodB* and *sodC* and the oxidoreductase gene *STM14_2022* was downregulated. Furthermore, the peroxidase genes had increased expression in the *Salmonella* within *Acanthamoeba*. The expression of *ahpCF* was 25× higher within *Acanthamoeba* than the control culture (Table 1).

In the *Salmonella* within *Acanthamoeba*, expression of the genes encoding heat shock proteins (*dnaKJ*, *ibpAB*, *hslJ*, and *STM14_1509*), the small regulatory RNA RprA, the universal stress proteins (*uspB* and *uspE*), and cold shock protein (*cspE*) was downregulated. Expression of *yjeY*, which encodes carbon starvation response, was downregulated within *Acanthamoeba*.

There were no significant differences between treatments in terms of their global expression regulator *rpoS* activity levels. The same was true for mammalian phagocytes [15,16,17].

### 2.4. Expression of Genes Associated with Virulence

Most of the genes responsible for virulence in *Salmonella* are located in the SPI regions. Of these, SPI-1 and SPI-2 are the largest. SPI-1 is considered critical for epithelial cell invasion. Within *Acanthamoeba*, most *Salmonella* operons, including those encoding T3SS-1, had increased expression. The genes such as *invH*, *invABEFG*, *prgKJIH*, and *orgABC* controlling *Salmonella* invasion in host cells and *sptP* involved in post-infection host cytoskeleton recovery [26] were significantly upregulated in the *Salmonella* within *Acanthamoeba* (Table 1). Expression of *sopE* encoding the SopE effector protein translocated by the SPI-1 T3SS was also upregulated in the internalized *Salmonella*. The SPI-1-encoded operon *spaSRQ* comprises needle complex genes that were significantly downregulated in the *Salmonella* within *Acanthamoeba*.

Expression of most SPI-2 operons including *ssaBCDE*, *sseABCDE*, *sscA*, *ssaMVN*, and *ssaRSTU* were downregulated in the *Salmonella* within *Acanthamoeba* (Table 1). SPI-5 encoded genes *pipC* and *sopB* were upregulated within *Acanthamoeba*. These genes have been shown to be crucial for invasion in host cells [27].

Six virulence plasmid operons involved in conjugative transfer including *traJYALEKBP*, *trbD*, and *traV* had downregulated expression in the *Salmonella* within *Acanthamoeba*.

Several genes located on pathogenicity islands and involved in infection had upregulated expression in the *Salmonella* within the *Acanthamoeba* SCV; for example, the expression of *ybdA* encoding the enterobactin (siderophore) exporter (EntS) was >16× higher than it was in the control libraries. Certain genes involved in multidrug resistance such as the efflux pump operon *mdlAB* were highly upregulated in the *Salmonella* within *Acanthamoeba*. Expression of lysozyme inhibitor gene *mliC* was downregulated in the *Salmonella* within the amoebae.

Expression of the genes *bssR* and *bssS* encoding biofilm regulatory proteins and *bsmA* encoding biofilm peroxide resistance protein were downregulated within *Acanthamoeba*.

Expression of the operons *fliLMNOPQR*, *fliDST*, and *flhCD* participating in flagellar biosynthesis and *motAB* and *cheAWM* responsible for flagellar motor and chemotaxis were upregulated within *Acanthamoeba* (Table 1).

**Figure 2:**
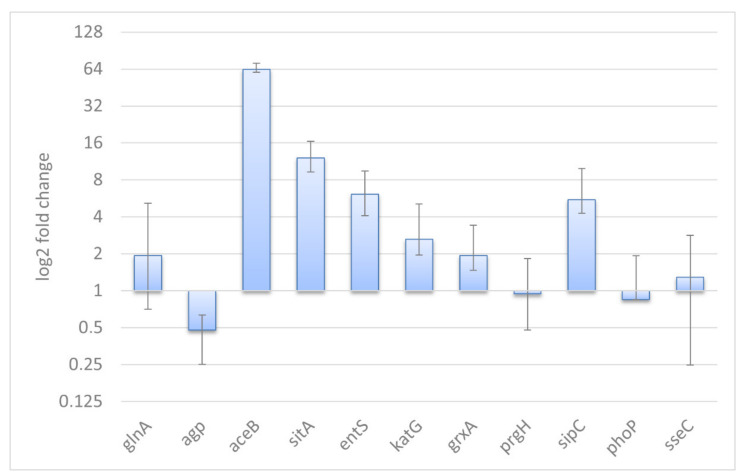
Relative expression levels of selected genes in *S*. Typhimurium. A value of 1 indicates no detectable difference in expression between intracellular *Salmonella* and control. Values > 1 indicate higher expression in intracellular bacteria and values < 1 indicate higher expression in control bacteria. Expression values normalized to the reference gene expression (*rpoD*).

Transcription of eleven genes (Table S4) in *Salmonella* and *Salmonella*–*Acanthamoeba* co-cultures was examined by qRT–PCR in another series of experiments. Eight genes, responsible for SPI-1, oxidative stress response (*grxA*, *katG*), iron uptake, and glucose-1-phosphatase were found to express in the same manner as it was observed in RNA-Seq analysis (Figure 2). Both Cappable-Seq and qRT-PCR data demonstrated that iron uptake and oxidative stress response genes were upregulated, while glucose-1-phosphatase gene was downregulated in *Salmonella* within *Acanthamoeba* compared to control *Salmonella* culture. Expression of *sipC* and *sitA* (both SPI-1 encoded) induced according to the qRT-PCR analysis, whereas expression of another gene encoded in SPI-1 (*prgH*) did not change. Besides, expression of SPI-2 encoded gene *sseC* was unaltered in contrast to downregulation found with RNA-Seq. Malate synthase gene (*aceB*) involved in GS cycle was significantly upregulated in SA samples based on the qRT-PCR data, which differs from the Cappable-Seq results (remains unchanged).

## 3. Discussion

### 3.1. Metabolic Pathways of Salmonella within Acanthamoeba SCV

**Figure 3:**
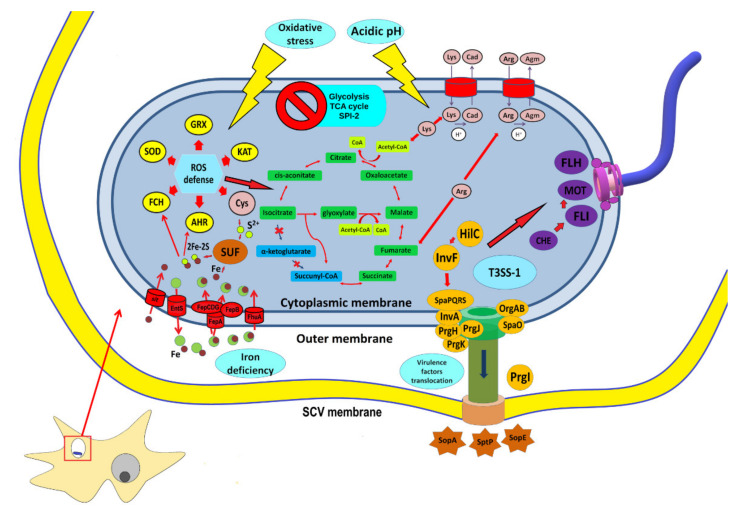
Reconstruction of the main metabolic and functional systems of *S*. Typhimurium responsible for *A*. *castellanii* infection. Enzymes involved in oxidative stress response are highlighted in yellow. Oxidoreductases included in Fe-S clusters (2Fe-2S) activating iron uptake system are in red (*ent*, *fhu*, and *fep*). Fe-S cluster assembly systems (SUF) are in brown. Glyoxylate shunt (GS; green) was shifted to the tricarboxylic acid (TCA) cycle and induced by reactive oxygen species (ROS). Low pH activates arginine (Arg) and lysine (Lys) metabolism (pink). Upregulation of SPI-1 genes (orange), flagellar apparatus (purple), and chemotaxis (purple) operons indicate that *Salmonella* penetrates protozoan cells. GRX, glutaredoxin 1; KAT, catalase G; AHR, alkyl hydroperoxide reductase; FCH, ferrochelatase; SOD, superoxide dismutase; SUF, Fe-S cluster assembly; Cys, cysteine; Lys, lysine; Cad, cadaverine; Arg, arginine; Agm, agmatine; FLH, flagellar assembly proteins flh; MOT, stator proteins MotAB; FLI, flagellar assembly proteins fli; CHE, chemotaxis.

Here, the TCA cycle, glycolysis, and the pentose phosphate pathway were downregulated in the *Salmonella* within *Acanthamoeba* (Figure 1) compared to those in the model macrophage and epithelial cell infections [15,17]. Glucose is considered a main carbon source during *Salmonella* infection [28]. Glucose, gluconate, and lactate are accessible to *Salmonella* inside mammalian phagocytes and are involved in the TCA cycle and glycolysis [17,29]. However, several studies showed that glyoxylate shunt (GS) is necessary for certain stages of *Salmonella* infection [30,31]. The glyoxylate cycle-related genes were upregulated in the *Salmonella* inside the *Acanthamoeba* SCV (Figure 3). The GS enables an organism to utilize acetate and fatty acids as carbon sources under physiological conditions requiring gluconeogenesis [32]. *Acanthamoeba* SCV may contain less glucose than mammalian macrophage SCV. Therefore, fast consumption of glucose in the protozoan SCV could trigger the switch from TCA and glycolysis to the GS. Downregulation of glycolysis gene expression, as well as upregulation of glyoxylate cycle genes was also confirmed by the qRT–PCR.

GS may also be implicated in *Salmonella* pathogenesis and oxidative stress response [32]. RNS formed in the phagosomes inhibit oxidative phosphorylation and TCA cycle genes [33]. Nitric oxide in phagosomes activates the iron citrate efflux transporter (IctE) that ferries citrate-chelated iron out of the cell [34]. Citrate is an intermediate metabolite in the GS and maintains iron homeostasis in *Salmonella* by chelating this metal [35]. Iron is an essential cofactor for several enzymes. However, excess iron may cause cell damage and is normally chelated by citrate [35]. Thus, GS increases the citrate pool required for iron neutralization and extracellular transport. Besides, in *Salmonella*, citrate export arrests growth and enhances oxidative stress resistance [34,35].

Expression of several operons encoding amino acid biosynthesis was upregulated in the *Salmonella* within *Acanthamoeba*. The cysteine, arginine, and lysine metabolic pathways were significantly enriched. Intermediates of the pathways may be involved in GS (Figure 3). Several amino acids play important roles in the adaptation of *Salmonella* to stress conditions. For example, cysteine biosynthesis was induced in *Salmonella* subjected to oxidative stress [36]. The arginine-dependent acid tolerance pathway was activated in *Salmonella* within *Tetrahymena* phagosomes [14]. Lysine is produced in response to acid stress through activation of the *cadAB* operon that provides lysine decarboxylation and cadaverine formation. Cadaverine undergoes protonation in acidic environments and raises the external pH [37]. Here, the arginine and lysine pathways might have been activated in response to the acidic environment of the *Acanthamoeba* SCV, whereas the cysteine pathway was upregulated in response to oxidative stress. Obviously, *Salmonella* engulfed by mammalian macrophages use similar protective pathways to acid stress like those in *Acanthamoeba* SCV, inducing cysteine and arginine biosynthesis [15,17]. In contrast, within epithelial cells, *Salmonella* downregulated expression of the *cadAB* operon [15].

LPS and its components (lipid A, O-antigen, core) are considered crucial for *Salmonella* virulence, macrophage survival, and acid stress response [38,39]. Upregulation of the operons involved in LPS and glycerophospholipid biosynthesis was observed in the *Salmonella* within *Acanthamoeba*. Similar findings were reported for *Salmonella* in *Dictyostelium discoideum* [12] and humanized mice [39]. Thus, the activation of LPS, glycerophospholipid, arginine, and lysine biosynthesis constitute the complex response of *Salmonella* to acidic conditions’ survival in protozoan and mammalian SCV.

### 3.2. Iron Uptake and Oxidative Stress

Iron is an essential growth factor for pathogenic bacteria [40] and a signaling element regulating virulence-associated genes [41].

Expression of the main operon associated with iron consumption (*sitABCD*) was upregulated in *Salmonella* within *Acanthamoeba* (Figure 3). A similar finding was reported for *Salmonella* phagocytosed by mammalian cells [15,17]. Moreover, in the *Salmonella* of the present study, iron uptake may have been mediated by additional ways. Thus, we observed the activation of the siderophore enterobactin *entS* and the Fe^3+^-catecholate (*fepA*, *fepB*, and *fepCDG*) and Fe^3+^-hydroxamate (*fhuACDB*) transporters (Figure 3). The iron acquisition systems are activated under iron-depleted conditions [42], and are essential for virulence of *Salmonella* and its survival in macrophages [43,44]. Thus, *Salmonella* within the *Acanthamoeba* SCV may undergo significant iron deficiency.

Iron determines the activity of certain regulatory and virulence genes [41,42]. Expression of genes encoding T3SS, flagellin, cold-shock protein (*cspE*), enterobactin transporter (*entS*), and other regulatory genes were upregulated under iron-depleted conditions [42]. Here, we observed similar changes in the transcriptome of *Salmonella* in the *Acanthamoeba* SCV presented with activation of flagellin biosynthesis and T3SS (Figure 3).

Iron is vital in the functioning of oxidoreductases that neutralize ROS such as hydrogen peroxide, superoxide, and hydroxyl radicals [45]. Both mammalian macrophages and *D. discoideum* amoeba utilize NADPH oxidase to generate bactericidal superoxide [46]. Phagocytosed bacteria respond to oxidative stress by producing catalases, peroxiredoxins, and superoxide dismutases that neutralize ROS [47]. In the present study, expression of several oxidoreductase genes was upregulated in response to oxidative stress, including *grxA*, *katG*, *sodA* (Mn-cofactored superoxide dismutase), and *ahpCF* (alkyl hydroperoxide reductase system). The *grxA* and *katG* genes were also activated in *Salmonella* phagocytosed by *Tetrahymena* [14] and macrophages [17]. In contrast, expression of *sodB* and *soxR* was downregulated in *Salmonella* inside *Acanthamoeba*. The sodA senses hydrogen peroxide whereas *sodB* and *soxR* detect superoxide-generating compounds [48]. Hence, H_2_O_2_ might be the main ROS produced by the amoebae. In addition, enzyme encoded by *katG* is able to inactivate RNS due to peroxynitritase activity [49].

Another mechanism of bacterial killing is based on Zn^2+^ and Cu^2+^ accumulation in phagosomes of mammals [50,51] and protozoa [52]. Zn^2+^ and Cu^2+^ attack Fe-S clusters vital to bacterial survival [52]. In the present study, the zinc-dependent, two-component system ZraRS responding to envelope stress was activated [53]. Perhaps, *A. castellanii* accumulate Zn^2+^ in its SCV to kill phagocytosed bacteria. Fe–S cluster-containing proteins are involved in many essential cellular processes, including overcoming the adverse effects of oxidative stress [54]. In *Salmonella*, Fe-S cluster assembly and delivery are controlled mainly by iron sulfur cluster (ISC) and sulfur mobilization (SUF) operons. The isc operon might regulate the housekeeping Fe–S cluster assembly system, whereas the suf operon controls Fe–S cluster biogenesis under iron starvation and oxidative stress [55]. Here, the *suf* operon was upregulated in phagocytosed *Salmonella*. Thus, upregulation of several genes encoding antioxidant defense factors in response to oxidative stress inside the amoebae protects *Salmonella* from protozoan bactericidal systems and potentiates bacterial virulence. Within *Acanthamoeba* SCV, *Salmonella* induces iron uptake systems participating in oxidative stress response, augmenting bacterial virulence, and establishing and maintaining iron homeostasis. Iron uptake activation may enhance bacterial resistance to the host Zn-dependent killing mechanism by increasing the iron concentration.

In this study, there was no upregulation of the *Salmonella* genes (*phoPQ*, *rpoE*, *soxS*, and *oxyR*) encoding factors responding to low osmolarity, low Ca^2+^ concentrations, or acidic pH. In contrast, the aforementioned genes were upregulated in macrophage SCV [17]. Expression of genes, encoding heat shock (*ibpAB*, *dnaKJ*), cold shock (*cspE*), osmoregulation (*ompW*), and universal stress response (*uspB* and *uspE*) were significantly downregulated in /the *Salmonella* within *Acanthamoeba*. Moreover, we noted the activation of the *cpxP* transcriptional repressor of the two-component system *cpxRA* governing envelope stress response [56]. Therefore, the conditions inside *Acanthamoeba* SCV seem to be more advantageous to *Salmonella* survival than those inside macrophage SCV. In *Acanthamoeba* SCV, *Salmonella* is subjected mainly to oxidative stress, whereas the bacterium would be exposed to numerous simultaneous stressors in macrophages.

### 3.3. Virulence and Motility

Recent studies reported the induction of several virulence genes in *Salmonella* interacting with protozoa [12,14,21]. The involvement of these genes in mammalian epithelial cell, fibroblast, and macrophage infection has been previously described [15,17,20]. The genes responsible for the interaction between the bacterium and host cells are combined into clusters collectively named SPI [28,57]. Here, we observed the upregulation of the SPI-1-encoded virulence factors *invH*, *invABEFG*, *prgKJIH*, and *orgABC* in *Salmonella* inside *Acanthamoeba* SCV. Induction of T3SS-1 encoded by SPI-1 is required to enable *Salmonella* to invade epithelial cells [58]. Earlier reports suggest that *Salmonella* invade *Acanthamoeba* by a mechanism similar to that for epithelial cells [15,22].

In the present study, in intracellular *Salmonella,* several genes encoding virulence factors in non-SPI regions were upregulated such as *srfC*, *ybjX*, *hupA*, and *msgA* encoding the putative virulence effector, VirK-like, HU DNA-binding, and virulence MsgA proteins, respectively. These proteins are involved in internalization and protection of *Salmonella* in epithelial cells [59,60] and macrophages [61].

It should be noted that in phagocytosed *Salmonella,* expression of SPI-2 genes associated with long-term persistence such as *ssaBCDE*, *sseABCDE*, *sscA*, *ssaMVN*, and *ssaRSTU* remain at low level. SPI-2 is activated in response to PO_4_^3−^, Mg^2+^, and Ca^2+^ deficiency, low pH and osmolarity within epithelial cells, macrophages [15,17], *Tetrahymena* phagosomes [14], and *Acanthamoeba* SCV [22]. However, Karlinsey et al. [39] showed that SPI-2 genes in *S.* Typhi were not required for mouse and macrophage survival during the early infection stages. Besides, it has been clearly shown on the *S*. Typhimurium infection of fibroblasts [20] that SPI-2 is not upregulated during first two h p.i. However, non-activated SPI-2-encoded genes suggest that *Salmonella* in *Acanthamoeba* SCV were unaffected by acid pH, low osmolarity, and Mg^2+^, Ca^2+^, and PO_4_^3−^ deficiency at early stage of infection. The latter two ions are major SPI-2 activation factors [62].

Free-living *Salmonella* upregulate genes governing flagellar assembly and flagellin synthesis in conditions conducive to active movement and adhesion. These genes are downregulated in macrophages and fibroblasts [20]; however, for unknown reasons, they are upregulated in epithelial cells at the early stage of infection [15]. Here, we detected induction of the operons controlling flagellum biosynthesis (*fliLMNOPQR*, *fliDST*, and *flhCD*), flagellar motor (*motAB*), and chemotaxis (*cheAWM*) in *Salmonella* within *Acanthamoeba*. Moreover, we found upregulation of the *tdcA* transcriptional activator in the *tdc* operon governing threonine and serine transport and metabolism during anaerobic growth [63]. The requirement of threonine and serine for flagellin biosynthesis and virulence was demonstrated via *Salmonella* Δ*tdcA* mutants, which were less virulent than the WT in mice [63,64].

### 3.4. Validation of the RNA-Seq Results and General Remarks

A new series of experiments was conducted with the same conditions for biological validation of the RNA-Seq results with qRT-PCR. Although, most genes demonstrated the same expression patterns, we should note a few discrepancies in the Cappable-Seq and qRT-PCR results. Particularly, SPI-1 encoded *prgH* showed unchanged expression in qRT-PCR, while it was upregulated in Cappable-Seq. Nevertheless, other two SPI-1 encoded genes, *sipC* and *sitA*, were upregulated significantly according to the qRT-PCR data that supports whole SPI-1 activation. Such contradictory trends in gene expression are in good agreement with non-simultaneous activation of SPI-1 genes reported by Ibarra et al. 2010 [65]. Expression of SPI-2 encoded *sseC* was unchanged based on the qRT-PCR analysis, while it was downregulated according to the Cappable-Seq data. This inconsistency can be related to the fact that different series of experiments have been carried out for RNA-Seq and qRT-PCR analyses. Anyway, no induction of *sseC,* revealed based on both methods, is in good agreement that expression of SPI-2 was unchanged at early infection times [19,20]. Expression of *aceB* remained unchanged according to the RNA-Seq data, while qRT-PCR demonstrated significant upregulation of this gene, which is in good agreement with RNA-Seq data, demonstrating activation of glyoxylate cycle accompanied with high enrichment score of this metabolic pathway. In addition, expression of *aceAB* is known to be regulated independently from other GS cycle genes [66]. Moreover, the samples used for qRT-PCR were distinct to the samples used for RNA-Seq and that could be a source of the discrepancy seen in the validation tests. Except for those three genes, we found a good coincidence in the expression patterns for other eight genes based on the RNA-Seq and qRT-PCR data.

The transcriptome of the *Salmonella* in *A. castellanii* SCV significantly differed from those of the *Salmonella* in other protozoa [14,22] and mammalian phagocytes [15,17]. In fact, viability visibly differs among various types of phagocytic cell infected by the same bacterium. For example, *S.* Typhimurium is not cytotoxic to *Tetrahymena,* whereas it induces massive apoptosis-like death in *Acanthamoeba rhyzodes* at 24 h post-infection [14,22]. Transcriptomic data showed significant differences in the gene expression in *Salmonella* within protozoa of different taxa, e.g., far more SPI-1 genes were activated within *A. rhysodes* than *Tetrahymena* [14,22]. Genes controlling translation, ribosomal structure, and biogenesis were induced at 3 h post-infection within *Tetrahymena* but at 8 h post-infection in amoebae. O-antigen and T6SS are required for *Salmonella* to survive inside *D. discoideum* [12]. Expression of SPI-2 genes was unchanged in *Salmonella* within *A. castellanii*, but upregulated in the bacteria within *A. rhyzodes*. In contrast, the genes encoding flagellum biosynthesis were upregulated in *A. castellanii* but downregulated in *A. rhyzodes* [22]. These differences might be explained by dissimilar infection time points (5 h post-inoculation in *A. rhyzodes* but 2 h post-inoculation in *A. castellanii*) and different *Salmonella* strains (*S. choleraesuis* SC-B67 vs. *S.* Typhimurium 14028S). Obviously, during the long evolutionary history of the interactions between *Salmonella* and phagocytes, the bacteria could develop various mechanisms to ensure their survival and persistence in protozoa, serving as a “Trojan horses” for bacterial pathogens.

A limitation of our study is the evaluation of *Salmonella* transcriptome only at an early stage of *Acanthamoeba* infection. This stage of infection allows to disclose specific expression patterns that support survival of *Salmonella* adapting to the *Acanthamoeba* SCV conditions. However, *Salmonella* can change their expression patterns at a late stage of *Acanthamoeba* infection, which is accompanied by multiplication of the bacteria within SCV and host cell death [22]. Therefore, to understand molecular mechanisms of prolonged *Salmonella* persistence in protozoan cells, the study of a time-dependent transcriptional adaptation is required. However, such study may be complicated by massive apoptosis-like cell death induced by *Salmonella* at later stages of the infection [22].

**Figure 4:**
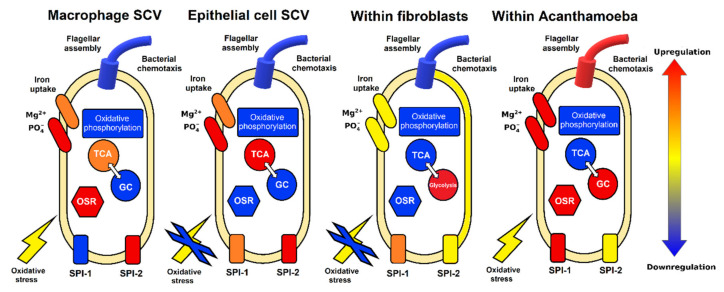
Response of *S*. Typhimurium to intracellular environment of macrophages [15,17], epithelial cells [15], fibroblasts [20], and *A*. *castellanii* (this study). Each symbol represents functionally related proteins. Colors reflect the expression levels of appropriate genes under each condition. Oxidative stress response, OSR; Glyoxylate cycle, GC; tricarboxylic acid cycle, TCA.

In this study, we evaluated the response of *Salmonella* to conditions within *Acanthamoeba* at the early stage of infection. The transcriptional profile of *Salmonella* in *Acanthamoeba* had certain similarities to those for *Salmonella* in macrophages, epithelial cells, and fibroblasts (Figure 4).

*Salmonella* are exposed to ROS in both macrophages and *Acanthamoeba*, but not in epithelial cells and fibroblasts. *Salmonella* are iron-deficient in both macrophages and *Acanthamoeba*. In response to intracellular conditions, the bacteria induce iron transport, oxidoreductase systems, and LPS biosynthesis. The *Acanthamoeba* model has similarities with the fibroblast infection model [20] by no-induction of SPI-2 and TCA cycle downregulation. In contrast to the macrophage model, SPI-1 expression was upregulated in the Acanthamoeba model, in epithelial cells and fibroblasts. Unlike known bacterial infection models in macrophages and epithelial cells, *Salmonella* in *Acanthamoeba* SCV at 2 h p.i. presented with specific patterns of gene expression, including switching from the TCA cycle and oxidative phosphorylation to the GS, activation of chemotaxis system, and flagellar apparatus biosynthesis (Figure 4). The gene expression profile of *Salmonella* in *Acanthamoeba* may reflect the symbiotic association between these organisms. The results of this study provided theoretical and empirical evidence of *Salmonella*-specific metabolic and virulence patterns for intracellular survival in unicellular and multicellular eukaryotes.

## 4. Materials and Methods

### 4.1. Strains

*Salmonella enterica* subspecies *enterica* serovar Typhimurium strain ATCC^®^ 14028 was obtained from the American Type Culture Collection (ATCC, Manassas, VA, USA) grown in Luria broth at 37 °C without antibiotics.

Axenic culture of free-living *Acanthamoeba castellanii* Neff strain ATCC^®^ 30010TM was obtained from the American Type Culture Collection (ATCC, Manassas, VA, USA). Trophozoites were cultivated in 10 mL of peptone-yeast extract-glucose medium (PYG; 2% (*w*/*v*) proteose peptone, 0.1% (*w*/*v*) yeast extract, 4 mM MgSO_4_ × 7H_2_O, 0.5 mM CaCl_2_, 0.1 M sodium citrate × 2H_2_O, 2.5 mM Na_2_HPO_4_ × 7H_2_O, 2.5 mM KH_2_PO_4_, 0.05 mM Fe(NH_4_)_2_(SO_4_)_2_ × 12H_2_O, and 0.1 M glucose; pH 7.0) in sterile 50-mL conical tubes (Eppendorf AG, Hamburg, Germany) at 25 °C. Reseeding was performed every 7 d.

### 4.2. Co-Culture Conditions

*A. castellanii* axenic culture was grown for 5 d in PYG medium to a density of 4–5 × 10^5^ cells/mL. The cells were centrifuged at 800× *g* for 5 min at 25 °C, washed twice with Page’s Amoeba Saline (PAS; 1.6 mM MgSO_4_ × 7H_2_O, 0.4 mM CaCl_2_, 0.1% sodium citrate × 2H_2_O, 2.5 mM NaH_2_PO_4_, 2.5 mM K_2_HPO_4_, and 0.05 mM Fe(NH_4_)_2_(SO_4_)_2_ × 12H_2_O), and suspended in 10 mL fresh PAS. The washed cells were incubated for 1 h at 25 °C until they were adherent.

*S.* Typhimurium was grown in LB medium at 37 °C without shaking until the late logarithmic phase. The cells were collected by centrifugation at 3000× *g* for 5 min at 25 °C, washed twice with PAS, suspended in fresh PAS, and seeded at 25 °C for 30 min until they adapted to the new growth conditions. The *Salmonella* cells were added to the *Acanthamoeba* culture in sterile PAS at a 100:1 bacterium:amoeba ratio. The co-culture was incubated until most of the bacteria had been ingested by the *Acanthamoeba*. At 1 h from the start of co-incubation, the suspension was centrifuged at 800× *g* for 10 min at 25 °C. Supernatants were discarded (for 1 min). Then three cycles of washing were carried out. Each cycle included next steps: pellets were resuspended with gentle shaking in sterile PAS for 1 min and incubated for 4 min at 25 °C; infected amoeba cells were centrifuged at 800× *g* for 10 min at 25 °C; supernatants were discarded (for 1 min). Final pellets were fixed in 1 mL ExtractRNA reagent (Evrogen, Moscow, Russia) exactly in 2 h from the start point of the infection.

**Figure 5:**
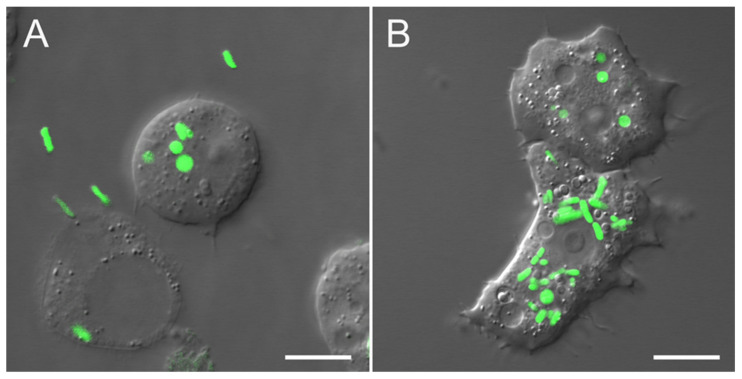
*Acanthamoeba castellanii* infected with *S.* Typhimurium (GFP) before (**A**) and after (**B**) washing procedures. Confocal laser scanning microscopy: differential interference contrast overlays with single optical GFP fluorescence sections in the green channel. Scale bars: 10 µm.

Confocal microscopy confirmed that after washing, most *Acanthamoeba* cells contained visible *Salmonella* cells inside the cytoplasm (Figure 5). To control extracellular *Salmonella* cells, the supernatants after the last washing were examined with LB plating and PCR. One hundred μL of supernatants were inoculated on the LB plates and spread with a sterile glass spatula. After incubation at 37 °C for 72 h, CFU were not detected. Control of extracellular *Salmonella* was also performed using PCR (Figure S3). For PCR, the supernatant after each centrifugation was collected in a sterile 1.5 mL tube, then 100 μL was transferred to a new sterile 1.5 mL tube. Lysing matrix E (Zymo Research, Irvine, CA, USA) in equal volume was added, and the samples were homogenized in a Tissue Lyser LT (QIAGEN, Hilden, Germany) for 5 min at 50 Hz. Lysates were inactivated by heating at 95 °C for 5 min. PCR was conducted using a thermocycler (T100 Thermal Cycler, Bio-Rad, Hercules, CA, USA) in a 25 μL reaction mixture containing 3.75 μL of 5× Q5 reaction buffer (New England Biolabs, Ipswich, MA, USA), 1.25 μL of 2.5 mM dNTPs mix, 0.5 μL of each primer (10 mM), 0.2 μL of Q5 High-Fidelity DNA Polymerase (New England Biolabs, Ipswich, MA, USA), and 2 μL of template DNA. The cycling conditions were as follows: 5 min at 95 °C, followed by 35 cycles at 95 °C for 30 s, 60 °C for 30 s, and 72 °C for 30 s, and a final extension at 72 °C for 10 min. The PCR products were visualized using capillary electrophoresis in QIAxcel Advanced System (Qiagen, Hilden, Germany) with QIAxcel DNA Fast Analysis Cartridge and QX DNA size marker 15 bp–3 kb (Figure S3). The PCR product sizes were estimated using the QIAxcel ScreenGel Software (Qiagen, Hilden, Germany). The capillary electrophoresis clearly showed that amplicons are not detected after the third washing (Figure S3).

A control *Salmonella* was cultured under the aforementioned conditions, with certain modifications. The bacteria were grown for 2 h at 25 °C in sterile PAS in the absence of *Acanthamoeba* and washed thrice by centrifugation at 1200× *g* for 5 min at 25 °C.

### 4.3. Microscopy

*Salmonella* transformed with the eGFP-pBAD plasmid (a gift from Michael Davidson, Addgene plasmid #54762; http://n2t.net/addgene:54762; RRID:Addgene_54762) was used to confirm intracellular localization. Images of infected cells were acquired every hour using a Zeiss LSM 780 laser scanning confocal microscope equipped with a Plan-Apochromat 40×/1.3 Oil DIC or Plan-Apochromat 63×/1.4 Oil DIC objective (Zeiss, Jena, Germany). Single mages and time-lapse series were acquired using the ZEN 2012 Black software (Zeiss). Differential interference contrast was used for visualization of living cells on a TPMT detector. GFP fluorescence in *Salmonella* was quantitated by excitation at 488 nm with the argon laser and an emission at 490–569 nm in the Channel mode.

### 4.4. RNA Extraction and cDNA Library Preparation

Total RNA was extracted with ExtractRNA reagent (Evrogen, Moscow, Russia). The RNA samples were treated with a TURBO DNA-free™ kit (Thermo Fisher Scientific, Waltham, MA, USA) following the manufacturer’s instructions. The RNA content was determined in a Qubit fluorometer (Thermo Fisher Scientific, Waltham, MA, USA). RNA integrity, purity, and concentration were assessed with a Bioanalyzer 2100 (Agilent Technologies, Santa Clara, CA, USA).

The Cappable-Seq procedure was performed as previously described [24].

Briefly, total RNA from *Salmonella* (5 μg) or *Salmonella* plus *Acanthamoeba* (10 μg) was capped with 3′-desthiobiotin-GTP using a Vaccinia capping system (New England Biolabs, Ipswich, MA, USA). The RNA was then purified 4× with RNA wash buffer on the RNA Clean and Concentrator-5 column (Zymo Research, Irvine, CA, USA) and eluted in 30 μL nuclease-free water.

Desthiobiotin-GTP-capped RNA was fragmented by polynucleotide kinase buffer and incubated for 5 min at 94 °C. The 3′-phosphates were removed from the fragmented RNA with polynucleotide kinase buffer and T4 polynucleotide kinase at 37 °C for 15 min.

Thereafter 40 μL of eluted RNA was added to 40 μL hydrophilic streptavidin magnetic beads (New England Biolabs, Ipswich, MA, USA). The RNA-bead mixture was incubated at 25 °C for 20 min. The RNA–bead mixture was washed twice on a magnetic rack in Buffer A followed by two washes in Buffer B. The biotin-eluted RNA was collected by placing the tube on the magnetic rack. Eluted RNA was cleaned with AMPure XP beads (Beckman Coulter, Brea, CA, USA). Here, we used a single enrichment round to reduce the proportion of rRNA to <50%. The second enrichment round dramatically decreased RNA yield and left insufficient material for library preparation.

In this study, 8% to 15% of all reads in the SA libraries were mapped to the *Salmonella* genome (Table S1), and the mRNA enrichment level was comparable to that reported in a previous study on the *Wolbachia* endosymbiont of *Brugia malayi* [67]. In our study, 30–50% of total reads were mapped to coding regions. In the control libraries, 38–49% of total reads were mapped primarily to the 16S and 23S rRNA genes (Table S3). In the intracellular *Salmonella* libraries, the proportion of ribosomal transcripts was ≤50% of all reads (Figure S1), and most of them comprised 5.8S eukaryotic rRNA.

The desthiobiotin caps were removed with Thermopol Buffer (New England Biolabs, Ipswich, MA, USA) and RppH (New England Biolabs, Ipswich, MA, USA) at 37 °C for 60 min. The reaction was terminated by adding 0.5 µL of 0.5 M EDTA and heating at 94 °C for 2 min. The RNA was collected on AMPure beads (Beckman Coulter, Brea, CA, USA) as previously described and washed and eluted in 6 µL low TE buffer. A NEBNext small RNA library prep kit (New England Biolabs, Ipswich, MA, USA) was used to generate Illumina sequencing libraries that were then amplified through 15 PCR cycles. All libraries were sequenced on an Illumina HiSeq 2000 (50-bp single-end reads; Illumina, San Diego, CA, USA) at the Joint KFU-Riken Laboratory of Kazan Federal University, Kazan, Russia.

All RNA-seq data discussed in this publication have been deposited in NCBI’s Gene Expression Omnibus and are available through GEO Series accession number GSE173638.

### 4.5. qRT-PCR Analysis

Biological validation of the transcriptomic analysis was provided by qRT-PCR using primer sets for eleven genes (*glnA*, *sitA*, *entS*, *prgH*, *sipC*, *phoP*, *sseC*, *grxA*, *katG*, *agp*, *aceB*). The primers were designed using Vector NTI 9.1.0 (Thermo Fisher Scientific, Waltham, MA, USA) (Table S4). cDNA was synthesized from 1 µg RNA of *Salmonella* control culture or 3 µg RNA of *Salmonella*-*Acanthamoeba* co-culture using the M-MuLV Reverse Transcriptase (New England Biolabs, Ipswich, MA, USA). Three biological replicates and three technical replicates were used for each sample of two different conditions (*Salmonella* and *Salmonella*–*Acanthamoeba*). qRT–PCR was performed on CFX connect real-time PCR detection system (Bio-Rad, Hercules, CA) and rpoD was used as a reference gene because it demonstrated most stability in our conditions (determined by geNorm among four potential genes) and had the narrowest range of variability according to the Cappable–Seq analysis. A melting curve analysis (55 °C to 95 °C) was performed after the thermal profile to ensure specificity. PCR efficiency was calculated from the log-linear portion of the standard curves. Differential gene expression and fold changes were determined using REST 2009 software (https://www.gene-quantification.de/rest-2009.html (accessed on 6 July 2021)).

### 4.6. Bioinformatic Analysis

Demultiplexed reads were trimmed against adapters with bbduk (https://sourceforge.net/projects/bbmap/ (accessed on 16 February 2021)). SortMeRNA v.2.1 [68] was used to remove reads belonging to prokaryote and eukaryote rRNA sequences. Clean reads were initially mapped against the full *A. castellanii* genome (GCF_000313135.1) with Bowtie2 [69] in local mode (L-16) and then mapped against the *S.* Typhimurium 14028s genome (GCA_000022165.1) [70] using similar parameters. The mapped files were converted to *bam* format using SAMtools [71]. We found that 8% to 15% of all reads in SA libraries were mapped to the *Salmonella* genome (Table S1), and the mRNA enrichment level was comparable to that reported in a previous study on the *Wolbachia* endosymbiont of *Brugia malayi* [68]. Proportion of total reads primarily mapped to coding regions accounted 51–62% for the control *Salmonella* libraries and ≥50% for the intracellular *Salmonella* libraries (Table S3, Figure S1).

TSS were identified as previously described [24], with certain modifications. A 0.5–30.5 cutoff range was used in the bam2firstbasegtf.pl script (https://github.com/Ettwiller/TSS (accessed on 16 February 2021)). The number of revealed TSS was used to plot graphs and identify the optimal cutoff parameter. The TSS were clustered with the cluster_tss.pl script and cutoff of 5.

The genes in the RefSeq annotation divided the TSS into the categories InterS (intergenic TSS with downstream gene in same orientation), InterA (intergenic TSS with downstream gene in opposite orientation), IntraS (intragenic TSS in gene with same orientation), or IntraA (intragenic TSS in gene with opposite orientation) according to Boutard et al. [72]. The Bedtools toolset was used to search for the closest gene and determine its distance from the TSS [73]. Gene activity was determined as the total number of reads belonging to the TSS in the 300-bp gene (operon) region in the direct orientation of the corresponding open reading frame (ORF).

The reads per operon were evaluated with featureCounts in the Rsubread package of R [74]. Door2 database was used to identify genes in operons [75]. An operons and genes with the original read coverage scores were used for differential gene expression analysis in the DESeq2 package [76]. Genes with FDR < 0.05, read count > 10 and fold change > 2 were determined as significantly differentially expressed. A principal component analysis (PCA) plot was constructed and transformed by the regularized log approach in R. A metabolic pathway enrichment analysis was conducted in the fgsea package [77] against the KEGG database (https://www.kegg.jp/ (accessed on 18 February 2021)). The genes were assigned to COG functional category in eggNOG database v. 4.5 [78].

## Figures and Tables

**Table 1:** Selected operons and genes that were differentially expressed in *Salmonella* within *Acanthamoeba*.

Operon	Log_2_ Fold Change	Description
Metal uptake		
*sufABCDSE*	2.76	iron-sulfur cluster assembly
*fhu*	2.65	uptake of ferric hydroxamate
*ent*	3.55	enterobactin exporter
*fep*	1.23	ferric enterobactin transport protein
*fes*	2.73	ferric enterobactin esterase
*corA*	1.65	magnesium/nickel/cobalt transporter
Oxidative stress response		
*ahpCF*	3.67	alkyl hydroperoxide reductase
*grxA*	2.40	glutaredoxin A
*katG*	1.89	hydroperoxidase
*sodA*	2.18	superoxide dismutase (manganese)
*sodB*	−3.20	superoxide dismutase (iron)
SPI-1		
*sitABCD*	1.13	manganese/iron transport
*prgKJIH-* *orgABC*	1.27	needle complex
*hilA-iagB*	1.56	invasion protein
*spaPOL-invJI*	2.17	needle complex
*invABEFG*	1.20	invasion complex
*invH*	2.46	involved in the synthesis of the type III secretion system
SPI-2		
*ssaBCDE*	−2.36	type III secretion system apparatus
*sseABCDE*	−2.47	translocation machinery components
*ssaGHIJ*	−1.88	type III secretion system
*ssaMVN*	−2.66	type III secretion system
*ssaRSTU*	−1.88	type III secretion system
*ydhC*	1.68	inner membrane transport
Motility and chemotaxis		
*flh*	1.70	flagellar biosynthesis
*fli*	1.48	flagellar biosynthesis
*mot*	1.70	flagellar motor protein
*che*	1.70	chemotaxis

## Data Availability

All RNA-seq data discussed in this publication have been deposited in NCBI’s Gene Expression Omnibus and are available through GEO Series accession number GSE173638 (available online: https://www.ncbi.nlm.nih.gov/geo/query/acc.cgi?acc=GSE173638) (accessed on 22 August 2021).

## Supplementary Materials

The following are available online at https://www.mdpi.com/article/10.3390/ijms22169077/s1.
