# Supplementary material for: Cappable-Seq Reveals Specific Patterns of Metabolism and Virulence for Salmonella Typhimurium Intracellular Survival within Acanthamoeba castellanii
Source: Int J Mol Sci. 2021 Aug 23;22(16):9077. doi: 10.3390/ijms22169077 (PMC8396566; doi:10.3390/ijms22169077)
Supplement: Supplementary file 1 [file ijms-22-09077-s001.zip › Supplementary description.pdf]

Figure S1. Transcription start sites (TSS) identified in S. Typhimurium genome. Venn diagram showing TSS identified at 0 h and 2 h post-infection (p.i.) (a). Bar chart showing % TSS at 0 h (green) and 2 h p.i. (blue). classified as Inter_S, Intra_S, Inter_A, or Intra_A (b).

Figure S2. Gene set enrichment analysis performed using the fgsea package. Positive (normalized) enrichment scores indicate upregulation of bacterial genes in trial library pathway. Negative (normalized) enrichment scores indicate downregulation of bacterial genes in trial library pathway. Operon pathway annotation was determined against KEGG database.

Table S1. General RNA-Seq statistics and alignment report
Table S2. Statistics of TSS in Salmonella Typhimurium 14028S genome
Table S3. Differential expression of operons
