# Supplementary figures and images for: Cappable-Seq Reveals Specific Patterns of Metabolism and Virulence for Salmonella Typhimurium Intracellular Survival within Acanthamoeba castellanii
Source: Int J Mol Sci. 2021 Aug 23;22(16):9077. doi: 10.3390/ijms22169077 (PMC8396566; doi:10.3390/ijms22169077)

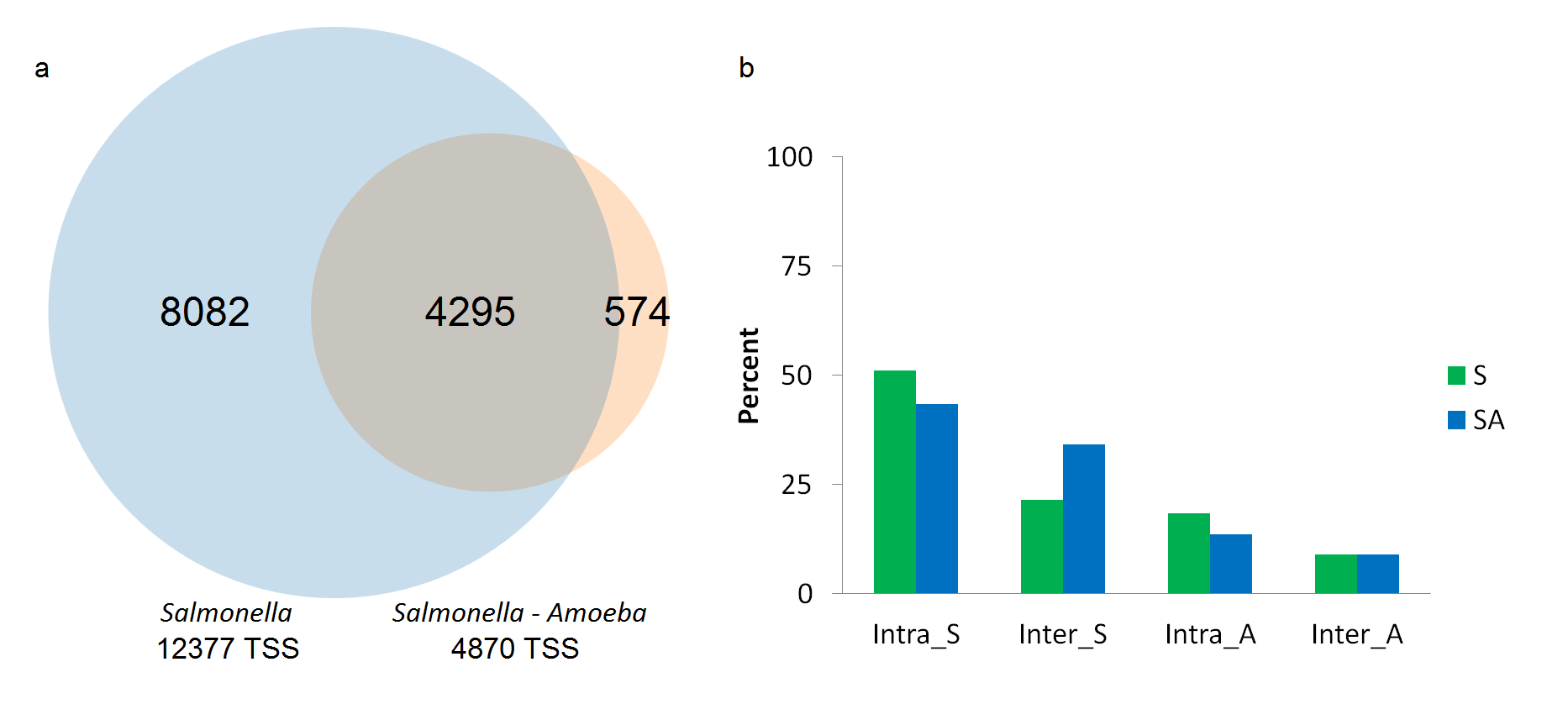

Supplement: Supplementary file 1 [file ijms-22-09077-s001.zip › Fig S1.tif]

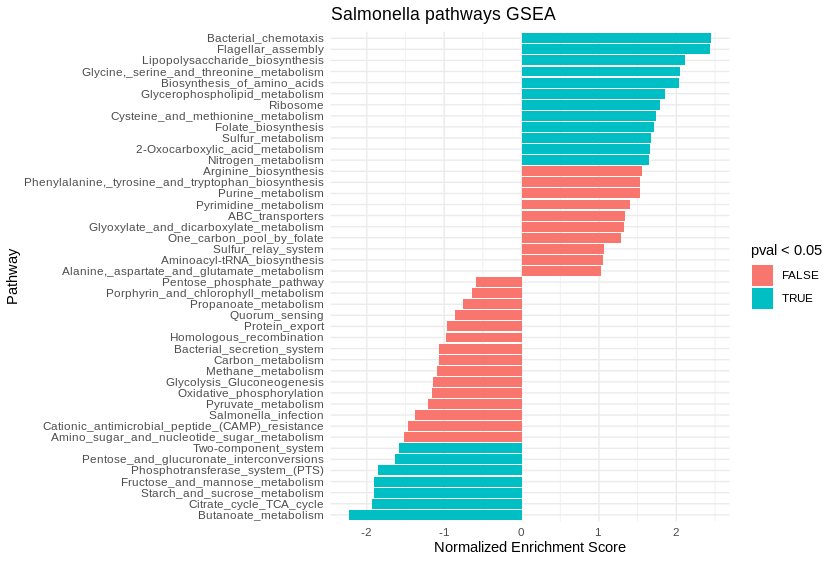

Supplement: Supplementary file 1 [file ijms-22-09077-s001.zip › Fig S2.tif]
